# Endoscopic submucosal dissection for a rare cause of esophageal mass: Von Recklinghausen's neurofibromatosis

**DOI:** 10.1055/a-2321-9697

**Published:** 2024-06-25

**Authors:** Huige Wang, Jiyu Zhang, Miao Shi, Bingrong Liu, Dan Liu

**Affiliations:** 1 191599 Department of Gastroenterology and Hepatology, The First Affiliated Hospital of Zhengzhou University, Zhengzhou, China

A 72-year-old woman was admitted to our hospital with a 20-year history of intermittent subxiphoid pain, which intensified in the past 2 months. Physical examination revealed dermal manifestations suggestive of neurofibromatosis ( Fig. 1 a). Enhanced computed tomography identified thickening of the distal esophageal wall. During upper endoscopy, a mass was observed stretching from the lower esophagus to the gastric cardia, leading to partial esophageal obstruction ( Fig. 1 b). Endoscopic ultrasound depicted a hypoechoic submucosal mass, distinguished by several anechoic lesions extending into the esophageal lumen ( Fig. 1 c). With informed consent from the patient, the mass was successfully excised using endoscopic submucosal dissection ( Fig. 1 d, Video 1 ). The extracted specimen measured 3.0 × 5.0 × 5.0 cm ( Fig. 1 e). Histopathological evaluation confirmed neurofibromatosis, identified by neurofibromatous cells encircling the specimen ( Fig. 1 f). The patient experienced no symptoms and showed no signs of recurrence or residual mass on follow-up endoscopic evaluations over 1 year.

**Fig. 1:**
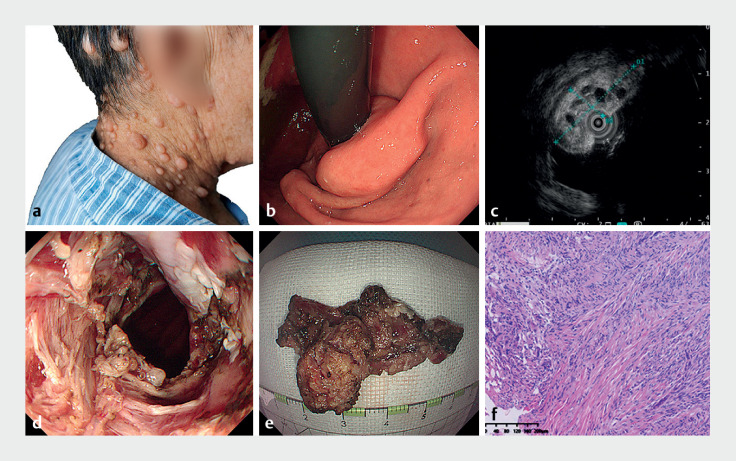
**a** Cutaneous manifestations of neurofibromatosis observed during physical examination. **b** Upper endoscopy showing a mass originating from the lower esophagus to the gastric cardia. **c** Hypoechoic, submucosally-originating mass with multiple anechoic projections into the esophageal cavity, as revealed by endoscopic ultrasound. **d** Complete removal of the mass via endoscopic submucosal dissection. **e** The resected specimen. **f** Histological analysis confirming the presence of neurofibromatosis.

Endoscopic submucosal dissection for a rare cause of esophageal mass: Von Recklinghausen's neurofibromatosis.Video 1

Von Recklinghausen's neurofibromatosis, an autosomal dominant condition, is generally characterized by dermal neurofibromas and distinctive café-au-lait spots. Though predominantly affecting the jejunum, stomach, ileum, and colorectum, esophagus involvement is scarce 1 2 . To the best of our knowledge, this is the inaugural report of an esophageal neurofibromatosis case managed with the ESD technique, demonstrating its safety and efficacy. This case underscores the need for endoscopists to be vigilant for signs of gastrointestinal neurofibroma, particularly in patients with dermal nodules, and suggests that endoscopic approaches like endoscopic submucosal dissection can provide an accurate diagnosis and effective treatment.

Endoscopy_UCTN_Code_TTT_1AO_2AG_3AD

## Notes

Endoscopy E-Videos https://eref.thieme.de/e-videos
*E-Videos* is an open access online section of the journal *Endoscopy* , reporting on interesting cases and new techniques in gastroenterological endoscopy. All papers include a high-quality video and are published with a Creative Commons CC-BY license. Endoscopy E-Videos qualify for HINARI discounts and waivers and eligibility is automatically checked during the submission process. We grant 100% waivers to articles whose corresponding authors are based in Group A countries and 50% waivers to those who are based in Group B countries as classified by Research4Life (see: https://www.research4life.org/access/eligibility/ ). This section has its own submission website at https://mc.manuscriptcentral.com/e-videos .

## References

[1] Tanaka M Kataoka H Joh T Neurofibroma of the esophagus complicating Von Recklinghausenʼs neurofibromatosis Am J Gastroenterol 2013 108 1935 1936 10.1038/ajg.2013.297 24300874

[2] Samat SH Onyemkpa C Torabi M Understanding esophageal neurofibroma: A case series and systematic review Int J Surg Case Rep 2020 76 450 457 10.1016/j.ijscr.2020.10.031 33207410 PMC7586048

